# Risk prediction model based on machine learning for predicting miscarriage among pregnant patients with immune abnormalities 

**DOI:** 10.3389/fphar.2024.1366529

**Published:** 2024-04-22

**Authors:** Yue Wu, Xixuan Yu, Mengting Li, Jing Zhu, Jun Yue, Yan Wang, Yicun Man, Chao Zhou, Rongsheng Tong, Xingwei Wu

**Affiliations:** ^1^ Department of Pharmacy, Personalised Drug Therapy Key Laboratory of Sichuan Province, Sichuan Academy of Medical Sciences and Sichuan Provincial People’s Hospital, School of Medicine, University of Electronic Science and Technology of China, Chengdu, China; ^2^ School of Pharmacy, Chengdu Medical College, Chengdu, China; ^3^ Department of Rheumatology and Immunology, Sichuan Provincial People’s Hospital, Chengdu, China; ^4^ Department of Gynaecology and Obstetrics, Sichuan Provincial People’s Hospital, Chengdu, China; ^5^ Department of Gastroenterology, Sichuan Provincial People’s Hospital, Chengdu, China

**Keywords:** immunological abnormality, pregnancy outcomes, machine learning, predictive models, clinical application

## Abstract

**Introduction:** It is known that patients with immune-abnormal co-pregnancies are at a higher risk of adverse pregnancy outcomes. Traditional pregnancy risk management systems have poor prediction abilities for adverse pregnancy outcomes in such patients, with many limitations in clinical application. In this study, we will use machine learning to screen high-risk factors for miscarriage and develop a miscarriage risk prediction model for patients with immune-abnormal pregnancies. This model aims to provide an adjunctive tool for the clinical identification of patients at high risk of miscarriage and to allow for active intervention to reduce adverse pregnancy outcomes.

**Methods:** Patients with immune-abnormal pregnancies attending Sichuan Provincial People’s Hospital were collected through electronic medical records (EMR). The data were divided into a training set and a test set in an 8:2 ratio. Comparisons were made to evaluate the performance of traditional pregnancy risk assessment tools for clinical applications. This analysis involved assessing the cost-benefit of clinical treatment, evaluating the model's performance, and determining its economic value. Data sampling methods, feature screening, and machine learning algorithms were utilized to develop predictive models. These models were internally validated using 10-fold cross-validation for the training set and externally validated using bootstrapping for the test set. Model performance was assessed by the area under the characteristic curve (AUC). Based on the best parameters, a predictive model for miscarriage risk was developed, and the SHapley additive expansion (SHAP) method was used to assess the best model feature contribution.

**Results:** A total of 565 patients were included in this study on machine learning-based models for predicting the risk of miscarriage in patients with immune-abnormal pregnancies. Twenty-eight risk warning models were developed, and the predictive model constructed using XGBoost demonstrated the best performance with an AUC of 0.9209. The SHAP analysis of the best model highlighted the total number of medications, as well as the use of aspirin and low molecular weight heparin, as significant influencing factors. The implementation of the pregnancy risk scoring rules resulted in accuracy, precision, and F1 scores of 0.3009, 0.1663, and 0.2852, respectively. The economic evaluation showed a saving of ¥7,485,865.7 due to the model.

**Conclusion:** The predictive model developed in this study performed well in estimating the risk of miscarriage in patients with immune-abnormal pregnancies. The findings of the model interpretation identified the total number of medications and the use of other medications during pregnancy as key factors in the early warning model for miscarriage risk. This provides an important basis for early risk assessment and intervention in immune-abnormal pregnancies. The predictive model developed in this study demonstrated better risk prediction performance than the Pregnancy Risk Management System (PRMS) and also demonstrated economic value. Therefore, miscarriage risk prediction in patients with immune-abnormal pregnancies may be the most cost-effective management method.

## 1 Introduction

Miscarriage is one of the most common pregnancy complications in obstetrics and gynaecology. In China, termination of pregnancy at less than 28 weeks of gestation with a foetus weighing less than 1,000 g is still defined as miscarriage (Writing Group Of Chinese Expert, 2020), and ESHRE defines miscarriage as pregnancy loss before 24 weeks of gestation (Bender et al., 2018). The maintenance and progression of pregnancy is a complex process governed by multiple developmental factors (Tasadduq et al., 2021). Pregnancy is associated with mechanisms that regulate the immune response at the maternal-fetal interface, and when pregnancy is combined with autoimmune abnormalities, the recurrence of autoimmune disease in some patients is associated with a significant incidence of adverse pregnancy outcomes (Ford and Schust, 2009; Robinson, 2014). Autoimmune diseases predispose to women of childbearing age (Chinese Society of Rheumatology of the Chinese Medical AssociationNational Clinical Research Center for Dermatologic and Immunologic DiseasesChinese Systemic Lupus Erythematosus Treatment and Research Group, 2020). aPLs is a general term for a group of autoantibodies that target phospholipids and/or phospholipid-binding proteins as antigens. aPLs in the diagnostic criteria include lupus anticoagulant (LA), anti-cardiolipin antibody (aPL), and anti-β2 glycoprotein 1 (β2-GP1) (Tektonidou et al., 2019). A retrospective study revealed approximately 9% aPL (positivity in patients with autoimmune diseases who experienced pregnancy loss (Han et al., 2017).In the group of patients with autoimmune diseases and autoantibody abnormalities, autoimmune abnormalities often increase the risk of adverse pregnancy outcomes (Carp et al., 2012). Systemic lupus erythematosus (SLE) is an autoimmune-mediated, diffuse connective tissue disease highlighted by immune inflammation (Chinese Medical Association Rheumatology Branch, 2010). Antiphospholipid syndrome (APS) is a non-inflammatory autoimmune disease that is characterised by arterial and venous thrombosis, morbid pregnancy (early miscarriage in pregnancy and stillbirth in mid-late pregnancy) and thrombocytopenia, and the presence of aPL, which may be present singly or in combination. The presence of aPL in the serum, which may be present singly or in combination (Chinese Medical Association Rheumatology Branch, 2011). Despite exceptions such as SLE or APS, the impact of immune abnormalities on reproductive health has been largely overlooked in clinical practice and research. Consequently, early and precise identification of patients at risk of miscarriage is crucial, necessitating timely intervention. Personalized medicine holds the potential to revolutionize the standard of care, shifting from generic guidelines to computational models based on individual patient data. This approach aims to develop more convenient diagnostic tools for women with immunologically abnormal pregnancies, accurately identify high-risk patients, and proactively intervene to support them in carrying their pregnancies to full term.

Currently, there are various criteria for assessing the risk of pregnancy in women in obstetrics. Scholars in England have created the Obstetric Early Warning Score tool (Singh et al., 2012).In 2017, the China Health and Family Planning Commission issued the Norms for the Assessment and Management of Maternal Pregnancy Risks (National Health and Family Planning Commission of the People’s Republic of China, 2017). Pregnancy risk management systems or early warning scoring systems are commonly used to assess the risk of pregnant patients in the past (Subbe et al., 2001; Jing et al., 2016; Coomarasamy et al., 2021). However, despite their use for risk assessment, these criteria have some limitations. For instance, they often fail to provide early warning of risk in early pregnancy. Moreover, the pregnancy risk management system relies on a risk classification system rather than a point system, which is hindered by individual differences and the subjective influence of the evaluator. This has led to insufficient clinical application, staffing and training challenges, and non-uniform development of healthcare (Practice Committee of the American Society for Reproductive Medicine, 2012; Ping et al., 2016; Zhang et al., 2023). The relationships between clinical characteristics and biomarkers in extensive studies are intricate and highly heterogeneous, making it challenging for clinicians to predict miscarriage risk using standardized scores. Consequently, there is still a lack of evidence in clinical practice to quantify the association of risk and risk outcomes, hindering the creation of miscarriage risk prediction tools that not only generate risk predictions but also provide interpretable rules to support clinicians’ understanding of the resulting risk pathways. Such tools could lead to improved diagnosis, treatment choices, and overall health system efficiencies.

In recent years, machine learning has been increasingly utilized to predict pregnancy outcomes in expectant mothers. By modelling information based on causal and/or statistical data, machine learning can potentially unveil hidden dependencies between environmental factors and diseases within extensive datasets (Bratic et al., 2018). Our systematic search of Pubmed for studies on machine learning applications in predicting pregnancy outcomes yielded 28 relevant studies. These studies demonstrated the use of machine learning algorithms such as Logistic Regression (LR), Artificial Neural Network (ANN), Random Forest (RF), eXtreme Gradient Boosting (XGBoost), and Support Vector Machine (SVM) to predict pregnancy outcomes (Lakshmi, 2016; Malacova et al., 2020; Khatibi et al., 2021; Vaulet et al., 2022). While these models incorporated various factors, including age, maternal history, gestational age, BMI, biomarkers, and immunological factors, several challenges and limitations persist.Firstly, some prediction models exhibited lower than optimal Area Under the Curve (AUC) values, indicating a more general prediction performance. Secondly, certain studies only accounted for single-factor variations, neglecting the potential impact of immune factors, underlying patient conditions, and other relevant variables on pregnancy outcomes. Additionally, some prediction models were excessively complex in their operational steps, consuming substantial resources, and although statistically acceptable, their results were challenging to interpret for clinical application. Nevertheless, the interpretability of unsupervised machine learning results and their seamless implementation in clinical practice remain crucial.Despite the significant advantages, the development of pregnancy outcome prediction models using advanced machine learning algorithms is still relatively uncommon. This presents a new frontier for health professionals and policymakers, emphasizing the need for computational methods based on large patient datasets to advance the field.

The study aimed to develop a predictive model for assessing the risk of miscarriage in patients with immunologically abnormal pregnancies, to pinpoint high-risk factors contributing to pregnancy loss, and to investigate the impact of fundamental patient characteristics, biomarkers, medication regimens, and underlying disease characteristics on pregnancy outcomes. By stratifying outpatients with a high risk of miscarriage, early warning can aid in the clinical management of patients. Moreover, identifying major risk factors can support clinicians in making informed medical decisions and implementing proactive pharmacological interventions, crucial for preventing or reducing the likelihood of miscarriage. Additionally, for low-risk patients, this can help minimize unnecessary treatment costs and shorten the duration of treatment, which is of utmost importance.

## 2 Materials and methods

### 2.1 Data acquisition

**FIGURE 1:**
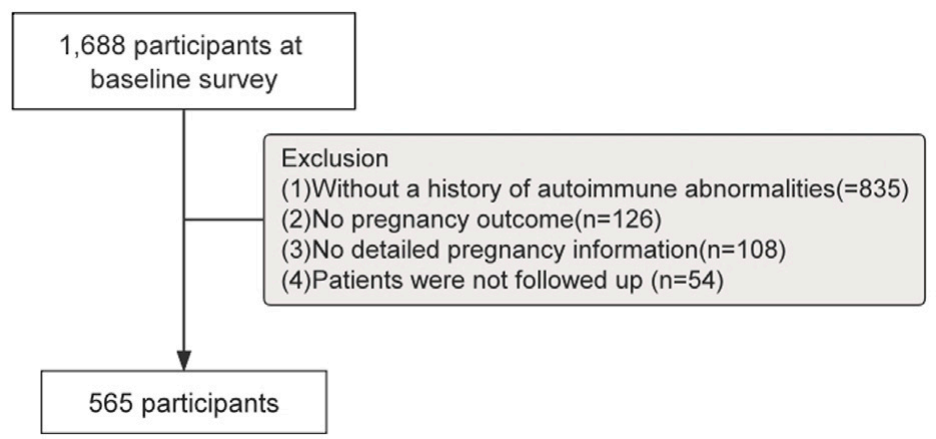
Details of patient inclusion-exclusion steps.

The prediction modelling was conducted at Sichuan Provincial People’s Hospital from October 2018 to October 2022, and we included all patients with complete demographic and clinical data from the Obstetrics and Rheumatology departments during this period. Out of 1668 samples. Medical data of these patients were retrospectively extracted from the electronic medical record (EMR) system. These medical data were generated and stored in the EMR system during diagnostic and laboratory tests. Medical data were generated and stored in the EMR system during diagnostic and laboratory testing. Face-to-face interviews were conducted with participating patients. Participants were selected based on the following criteria: 1) aged 18–45 years; 2) with a history of rheumatological immunity or abnormal autoantibody values; 3) on regular medication as prescribed by the doctor; and 4) with access to the patient’s complete course of treatment and pregnancy outcome. They were excluded if 1) treatment did not proceed as originally planned; 2) serious adverse reactions occurred during treatment; or 3) failure to obtain patient treatment and pregnancy outcomes. Miscarriage or spontaneous abortion resulting in foetal death before 20 weeks of gestation was defined as unintended termination of pregnancy. Ethical approval was obtained through the Ethics Committee of Sichuan Provincial People’s Hospital (Approval #2023-264). A detailed inclusion-exclusion flow chart is shown in Figure 1.

### 2.2 Research process of traditional evaluation methods

Biomarkers that are associated with the disease in the course of previous studies are scored, and before scoring is performed, a detailed history, physical and diagnostic screening programme is required, and according to the Maternal Pregnancy Risk Assessment Scale proposed by the National Health and Family Planning Commission of the People’s Republic of China (National Health and Family Planning Commission of the People’s Republic of China, 2017), the patient’s review is to include an assessment of rheumatological disease characteristics, and values are assigned according to each parameter, with a base score of 25% being given to yellow-risk-only entries, 50% being given to orange-risk and or yellow-risk entries, and 75% being given to red-risk entries, with details of the extra points given for each entry as shown in Table 1.

**TABLE 1:** Risk assessment rules.

Bonus points	Red risk entries	Orange risk entries	Green risk entries (%)
Basic marks			
Red risk entries (75%)	+5%	+2.5%	+1.25
Orange risk entries (50%)	—	+5%	+2.5
Green risk entries (25%)	—	—	+5

### 2.3 Cost-effectiveness analysis

The economic costs of the patient’s illness include direct costs: including direct medical costs and direct non-medical costs. 1) Direct medical costs: outpatient and emergency room costs, inpatient costs, retail drug costs, 2) Direct non-medical costs: other direct non-medical costs incurred by the patient and his/her companion such as travelling and nutritional costs for the visit to the doctor. By retrieving the details of outpatient and inpatient costs of 76 patients with immunologically abnormal pregnancies and 68 patients with miscarriages from the information department, an economic evaluation was conducted by calculating the average value of the costs. Transportation and nutritional costs were evaluated economically by telephone follow-up. Indirect costs: include the loss of labour productivity due to short-term and long-term disability and premature death, and the cost of lost labour for accompanying patients. The labour force population is defined statistically as the population in the 18–50 age group. The number of days of short-term and long-term incapacity and lost labour of companions is calculated for the current year only. Using the human capital method, the indirect economic burden refers to the economic impact on women resulting from pregnancy, which we evaluate based on productivity. According to the Chengdu Municipal Bureau of Statistics, the city’s GDP *per capita* in 2022 will be RMB 98,100 (Chengdu Daily, 2022). According to the Regulations on Population Planning and Maternity in Sichuan Province, the number of days of maternity leave is 158 days (Sichuan Daily, 2022).The methodology takes into account the different levels of productivity of each age group by giving it a certain weighting, with a productivity weighting of 0.75 for ages 15 to 49 (Jia et al., 1999). The approximate indirect cost of pregnancy is 31848.90. Details of the economics evaluation are shown in Tables 2, 3.

**TABLE 2:** Details of the cost of each type of disease.

Classifications	Outpatient costs	Hospital costs	Non-medical costs	Total costs
Live births patients	34780.42	16057.9	9617.09	60455.41
Low-risk patients	25871.93	10218.67	8435.43	44526.03
High-risk patients	43688.91	21897.13	10798.75	76384.79
Prevention costs 1	17816.98	11678.46	2363.32	31858.76
Miscarriages patients	3873.00	2299.75	2796.16	8968.90
Low risk - miscarriages	2420.05	1143.76	2265.72	5829.53
High risk - miscarriages	5325.94	3455.73	3326.59	12108.26
Prevention costs 2	2905.89	2311.97	1060.87	6278.73

**TABLE 3:** Details of the economics evaluation.

Pregnancy outcomes	Direct medical costs	Indirect medical costs	Total costs
Live births	60455.41	31848.90	92304.31
Miscarriages	8968.91	92304.31	101273.22

### 2.4 Model building process

#### 2.4.1 Data pre-processing

Data pre-screening consists of three steps: 1) removing variables with more than 90% missing data; 2) removing variables with more than 90% of individual values; and 3) removing columns with coefficients of variation less than 0.01. Any variable that meets the above criteria will be considered less informative and will be excluded.

#### 2.4.2 Data partition and dataset building

We utilized 80% of the data for training the model through random splitting, reserving the remaining 20% for testing the model’s performance. Inevitably, missing data occurred in practice. In cases where suspicious or missing data, including multiple missing values, were identified in the patient’s clinical characteristics section, the patient was contacted by telephone to rectify or supplement the information.

To mitigate the adverse effects of data imbalance on prediction performance, we employed two data sampling methods. These included the Synthetic Minority Over-Sampling Technique (SMOTE), which artificially generates new samples from underrepresented categories through interpolation, and the Support Vector Machine Synthetic Minority Over-Sampling Technique (SVMSMOTE), which utilizes Support Vector Machines (SVMs) to identify the samples used for generating new samples.

Feature selection was carried out using two methods. Firstly, the Least Absolute Shrinkage and Selection Operator (Lasso) was employed to penalize and discard unimportant variables (those with coefficients close to zero) by introducing penalty parameters through linear regression with L1 regularization, to evaluate the importance of variables and generate results. Secondly, Ridge regression (Ridge) was used, adding L2 regularized linear regression to limit the direction of change of the model coefficients, thereby minimizing the model coefficients and addressing the overfitting problem of the model. Variable importance was assessed based on the output of Lasso and Ridge (variable importance score), with a high score indicating that the variable improves prediction accuracy.

#### 2.4.3 Model development

By employing these two sampling methods and two feature selection techniques, we derived four datasets from the training set. Subsequently, we applied seven machine learning algorithms to each dataset, resulting in a total of 28 models. These included logistic regression (LR), Random Forest (RF), Content-Based Recommendations (CB), Support Vector Classifier (SVC), Multilayer Perceptron (MLP), Extreme Gradient Boosting (XGB), and K-nearest neighbour (KNN). These algorithms, well-suited for binary classification, were trained and applied to develop predictive models. Integrated algorithms have consistently demonstrated greater effectiveness and stability in predictive modelling compared to individual classification methods.Details regarding the parameters of the models developed using different algorithms are presented in Table 4.

**TABLE 4:** The detailed information of 4 datasets.

Number	Sampling methods	Screening methods	Number of variables	Number of train samples
1	SMOTE	Lasso	15	754
2	SMOTE	Ridge	8	754
3	SVMSMOTE	Lasso	15	537
4	SVMSMOTE	Ridge	8	537

### 2.5 Model explanation

Additionally, SHAP, a Python “model interpretation” package, was utilized to interpret the output of the machine learning models. Inspired by cooperative game theory, SHAP constructs an additive explanatory model considering all features as “contributors.” For each prediction sample, the model generates a prediction value, and the SHAP value represents the contribution of each feature in the sample. The impact of each variable on the predictive model was assessed through SHapley’s additive interpretation (SHAP).

### 2.6 Model evaluation

**FIGURE 2:**
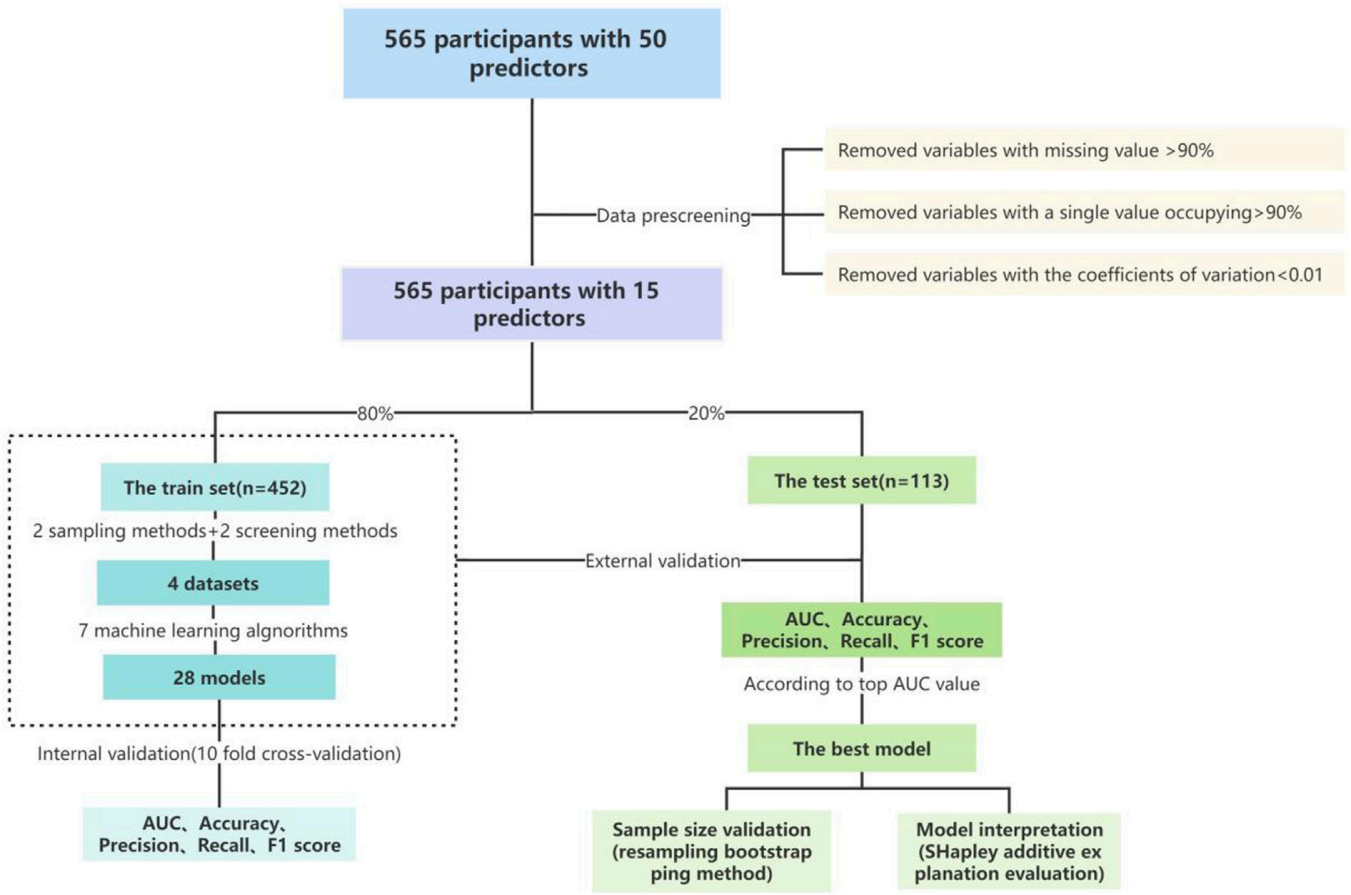
The main flow chart of the study.

The model underwent training in the training set to minimize the loss function. Internal validation was carried out using a 10-fold cross-validation method across 28 datasets, with 10 independent replicates collected between the metrics. Subsequently, external validation was conducted using the test set. The model’s prediction performance was assessed using various metrics, including the area under the receiver operating characteristic curve (AUC), accuracy, precision, recall, F1 score, Brier score, specificity, and area under the precision-recall curve (AUPRC). The best performing model was chosen as the predictive model.Furthermore, a multifactor analysis was executed to elucidate the combined contribution of different variables, sampling methods, screening methods, and machine learning algorithms. The details of the modelling process are depicted in Figure 2.

### 2.7 Sample size validation

The AUC of the best model was employed to evaluate the impact of sample size on model performance. The training set was partitioned into 10 sub-samples, with one sub-sample serving as validation data and the remaining nine sub-samples used for training. Cross-validation was repeated 10 times, each time with a different sub-sample, ensuring that the results were averaged or using other combinations to determine the optimal sample size.

### 2.8 Statistical analysis

Continuous variables were represented as means and standardized tables, while categorical variables were presented as frequencies and percentages. Statistical analysis was conducted using Stats in Python 3.8, and model development was carried out using Sklearn in Python 3.8.

## 3 Results

### 3.1 Population demographics

In summary, our study encompassed 565 patients, with 50 explanatory variables selected. These variables comprised four basic characteristic items, 12 pregnancy disorder items, 19 abnormal antibody value items, and 14 pregnancy medication items. Among the patients, adverse pregnancy outcomes were observed in 90 cases (15.93%), with 11 (12.22%) experiencing biochemical pregnancies, 63 (70.00%) encountering miscarriages, and 16 (17.78%) facing stillbirths. The mean age of the patients was 30.1 ± 4.1 years, and the average number of previous abortions was 1.4 ± 1.3. Additionally, 371 patients were found to have autoimmune diseases (65.7%). Detailed patient demographic and clinical information, serving as independent variables, and pregnancy outcomes, acting as the dependent variable, are presented in Table 5.

**TABLE 5:** The detailed information of participants.

Variable	Identifier	Parameter	Value(N=565)
**Basic characteristics**			
Age*	X1	N	565
		Mean±SD	29.7±3.8
		Median	30
		Minimum,maximum	18,50
Number of previous miscarriages*	X2	N	565
		Mean±SD	1.20±0.06
		Median	1
		Minimum,maximum	0,8
History of rheumatic immune disease*	X3	N	565
		Yes	371(65.7%)
		No	194(34.3%)
Number of underlying diseases*	X4	N	565
		Mean±SD	0.76±0.66
		Median	1
		Minimum,maximum	0,5
**Incidence of pregnancy complications**			
Number of complications during pregnancy*	X5	N	565
		0	287(50.8%)
		1	159(28.1%)
		2	85(15.1%)
		3	26(4.6%)
		4	8(1.4%)
**Gestational diabetes**	X6	N	565
		Yes	55(9.7%)
		No	510(90.3%)
**Thrombocytopenia**	X7	N	565
		Yes	43(7.6%)
		No	522(92.4%)
**Pregnancy induced hypertension**	X8	N	565
		Yes	35(6.2%)
		No	530(93.8%)
**intrahepatic cholestasis of pregnancy**	X9	N	565
		Yes	33(5.8%)
		No	532(94.2%)
**Lupus nephritis**	X10	N	565
		Yes	24(4.2%)
		No	541(95.8%)
**Hypothyroidism**	X11	N	565
		Yes	54(9.6%)
		No	511(90.4%)
**Abnormal liver enzymes**	X12	N	565
		Yes	29(5.1%)
		No	536(94.9%)
**Anemia**	X13	N	565
		Yes	38(6.7%)
		No	527(93.3%)
**Hyperlipidemia**	X14	N	565
		Yes	5(0.9%)
		No	560(99.1%)
**Thrombophilia**	X15	N	565
		Yes	11(1.9%)
		No	554(98.1%)
**Number of other pregnancy complications**	X16	N	565
		Yes	77(19.9%)
		No	309(80.1%)
**Abnormal antibody values**			
ANA*	X17	N	565
		0	314(55.6%)
		1:100	133(23.5%)
		1:320	46(8.1%)
		1:1000	40(7.1%)
		1:3200	32(5.7%)
Anti-SSA*	X18	N	565
		0	363(64.2%)
		+	79(14.0%)
		++	30(5.3%)
		+++	93(16.5%)
**Anti-SSB**	X19	N	565
		0	519(91.9%)
		+	22(3.9%)
		++	6(1.1%)
		+++	18(3.2%)
Anti-Ro52*	X20	N	565
		0	416(73.6%)
		+	43(7.6%)
		++	16(2.8%)
		+++	90(15.9%)
**Anti-nRNP/Sm**	X21	N	565
		0	495(87.6%)
		+	16(2.8%)
		++	18(3.2%)
		+++	36(6.4%)
**Anti-RNP**	X22	N	565
		0	496(87.8%)
		+	30(5.3%)
		++	6(1.1%)
		+++	33(5.8%)
**Anti-dsDNA**	X23	N	565
		0	509(90.1%)
		+	37(6.5%)
		++	9(1.6%)
		+++	10(1.8%)
**Anti-Sm**	X24	N	565
		0	529(93.6%)
		+	19(3.4%)
		++	10(1.8%)
		+++	7(1.2%)
**Anti-Jo1**	X25	N	565
		0	547(96.8%)
		+	14(2.5%)
		++	2(0.35%)
		+++	2(0.35%)
**Anti-Scl70**	X26	N	565
		0	562(99.47%)
		+	2(0.35%)
		++	1(0.18%)
**Anti-PM0Scl**	X27	N	565
		Positive	14(2.5%)
		Negative	551(97.5%)
**Anti-CENPB**	X28	N	565
		Positive	5(0.9%)
		Negative	560(99.1%)
**Anti-PCNA**	X29	N	565
		Positive	14(2.5%)
		Negative	551(97.5%)
**Anti-AMA/M2**	X30	N	565
		0	541(95.8%)
		+	16(2.8%)
		++	5(0.9%)
		+++	3(0.5%)
**AHA**	X31	N	565
		0	516(91.3%)
		+	28(5.0%)
		++	16(2.8%)
		+++	5(0.9%)
**ANuA**	X32	N	565
		0	535(94.7%)
		+	24(4.2%)
		++	4(0.7%)
		+++	2(0.4%)
**ACA**	X33	N	565
		Positive	62(11%)
		Negative	503(89%)
**Anti-β2GP1**	X34	N	565
		Positive	53(9.4%)
		Negative	512(90.6%)
**LA**	X35	N	565
		Positive	57(10.1%)
		Negative	508(89.9%)
**Medication during pregnancy**			
Number of medications used*	X36	N	565
		Mean±SD	3.23±2.34
		Median	3
		Minimum,maximum	0,15
HCQ*	X37	N	565
		Used	350(61.9%)
		Not used	215(38.1%)
GCs*	X38	N	565
		Used	332(58.8%)
		Not used	233(41.2%)
APC*	X39	N	565
		Used	292(51.7%)
		Not used	273(48.3%)
LMWH*	X40	N	565
		Used	191(33.8%)
		Not used	374(66.2%)
**TAC**	X41	N	565
		Used	61(10.8%)
		Not used	504(89.2%)
Prog*	X42	N	565
		Used	115(20.4%)
		Not used	450(79.6%)
**CSA**	X43	N	565
		Used	24(4.2%)
		Not used	541(95.8%)
**AZA**	X44	N	565
		Used	4(0.7%)
		Not used	561(99.3%)
**MMF**	X45	N	565
		Used	2(0.4%)
		Not used	563(99.6%)
**TNF- α inhibitor**	X46	N	565
		Used	8(1.4%)
		Not used	557(98.6%)
**G-CSF**	X47	N	565
		Used	4(0.7%)
		Not used	561(99.3%)
**Fat Emulsion**	X48	N	565
		Used	1(0.2%)
		Not used	564(99.8%)
**IVIG**	X49	N	565
		Used	13(2.3%)
		Not used	553(97.7%)
Number of other drugs*	X50	N	565
		Mean±SD	0.76±1.33
		Median	0
		Minimum,maximum	0,10

Note:(*)Represents important variables for modeling. The table is based on descriptive statistics of basic patient information, where continuous variables we analysed by Mean±SD, Median, Minimum, and maximum; dichotomous variables we analysed by percentage of Yes, No; multivalued ordinal variables in this study were only related to antibody value abnormalities, which were also expressed by percentage of each.

### 3.2 Implementation results of traditional methods

In terms of pregnancy risk scoring, a risk threshold of >25% was defined, resulting in 76 True Positives (TP), 94 True Negatives (TN), 381 False Positives (FP), and 14 False Negatives (FN). The accuracy of the traditional method, as indicated by Precision and F1 scores, was 0.3009, 0.1663, and 0.2852, respectively.

### 3.3 Economic evaluation

The actual cost can be calculated using the formula:

$$\text{Actual cost}=\left(\text{Number of abortions }*\text{ Cost of abortions}\right)+\left(\text{Number of live births }*\text{ Cost of live births}\right)$$

$$\text{Model cost}=\left(\text{TN }*\text{ live birth cost}\right)+\left(\text{FN }*\text{ abortion cost}\right)+\left(\text{TP }*\;\left(\text{prevention cost}2+\text{abortion cost}\right)\right)+\left(\text{FP }*\;\left(\text{prevention cost}1+\text{live birth cost}\right)\right)$$

Model value=Actual cost − model cost

Actual cost is 29,523,520.8. Based on the model, the values obtained are TN = 250, FN = 55, TP = 228, and FP = 32. The model cost can be calculated as follows: Model cost is 22,037,655.1. Therefore, the model value is calculated as Model value is 7,485,865.7. This indicates that pregnancy risk prediction is still likely to be the most cost-effective management method.

### 3.4 Dataset pre-screening

Following data pre-processing criteria screening, 35 variables were removed, leaving 15 variables retained for analysis. These retained variables encompassed age, history of previous miscarriages, rheumatological-immune disease comorbidities, number of underlying medical conditions, number of pregnancy complications, antinuclear antibodies, anti-SSA antibodies, anti-RO52 antibodies, total number of medications during pregnancy, hydroxychloroquine, glucocorticosteroids, aspirin, low-molecular heparin, progesterone, and number of other medications used.

### 3.5 Model evaluation

**FIGURE 3:**
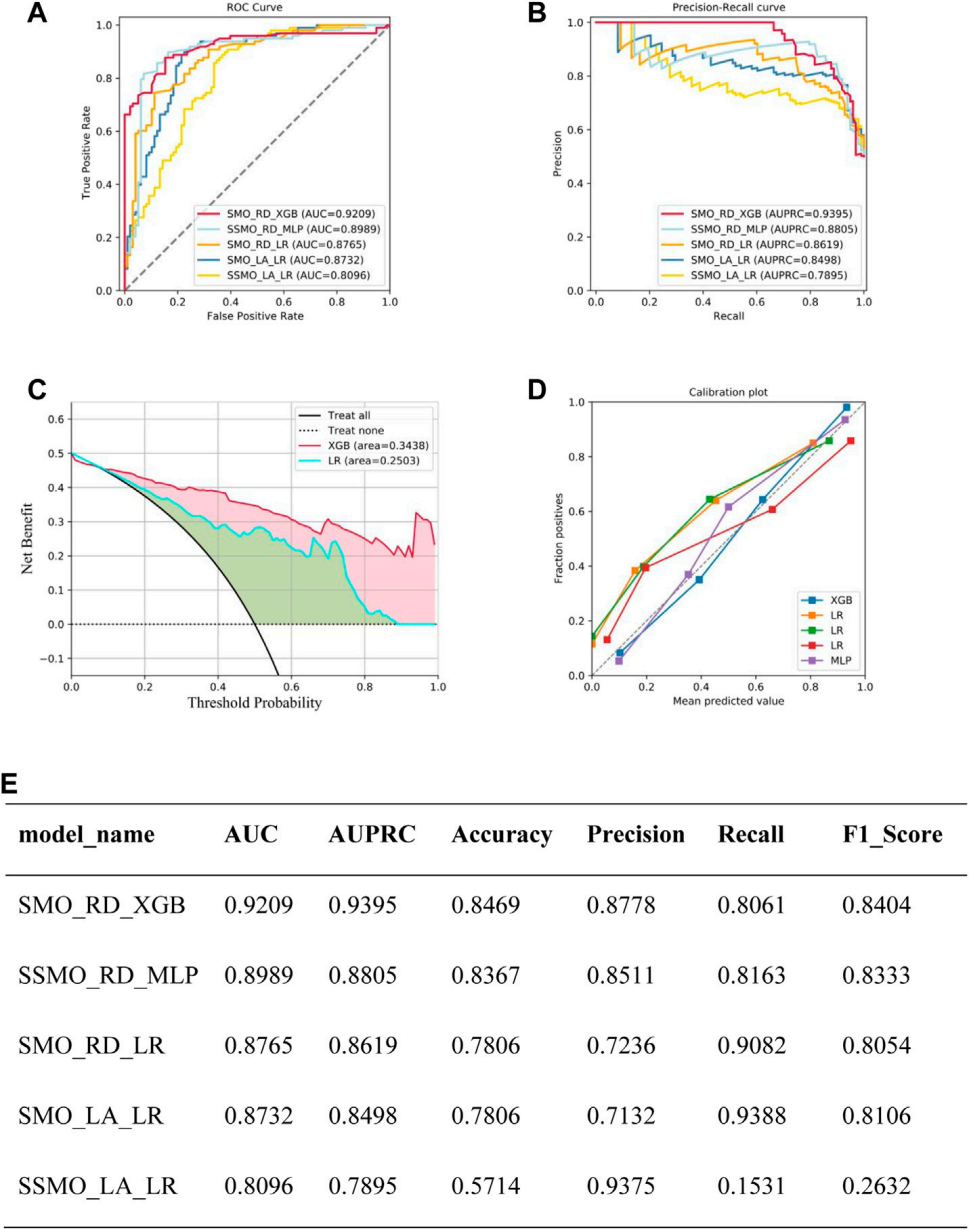
Summary of model performance: **(A)** Area Under the Curve (AUC) results for the top five models. **(B)** Precision-Recall (P–R) results for the top five models. **(C)** Decision Curve Analysis (DCA) results for the top five models. **(D)** Calibration curve results for the top five models. **(E)** Detailed performance metrics for the top five models.Note: SMO_RD_XGB means sampled by SMOTE, Ridge for feature selection, XGBoost as a model constructed by machine learning algorithm, SSMO_RD_MLP means sampled by SVMSMOTE, Ridge for feature selection, XGBoost as a model constructed by machine learning algorithm, SMO_RD_LR means sampled by SMOTE sampling, Ridge for feature selection, logistic regression as the model constructed by the machine learning algorithm, SMO_LA_LR meaning sampling via SMOTE, Lasso for feature selection, Logistic Regression as the model constructed by the machine learning algorithm, SSMO_LA_LR meaning Sampling through SVMSMOTE, Lasso for feature selection, Logistic Regression as a model constructed by machine learning algorithm.

A total of 28 models underwent validation in the test set, serving as external validation, and model performance metrics were generated. The top four models were identified based on their AUC values. The best-performing model (Model 1) was achieved by employing SMOTE as the sampling method, Ridge as the feature filtering method, and XGboost as the machine learning algorithm. This model exhibited AUC, AUPRC, Accuracy, Precision, Recall, and F1_score specificity values of 0.9209, 0.9395, 0.8469, 0.8778, 0.8061, and 0.8404, respectively. The parameter details of the models developed using different algorithms are presented in Figure 3.

### 3.6 Model explanation

**FIGURE 4:**
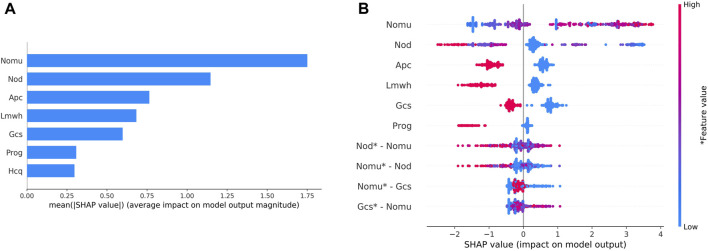
Variable contribution to the model by SHAP Value.**(A)** Contribution of each feature value in one sample. **(B)** Summary of SHAP value of each variable. Note: “Nomu” represents the total number of medications used in pregnancy, “Nod” represents a total number of other medications, “Apc” represents aspirin use in pregnancy, “Lmwh” represents low molecular heparin use in pregnancy, “Gcs” represents glucocorticoid use in pregnancy, and “Prog” represents glucocorticoid use in pregnancy. “Hcq” stands for hydroxychloroquine in pregnancy.

During external validation, the predictive models were evaluated based on their SHAP values, as depicted in Figure 4A. The wider the blue area, the greater the influence of the variable on the result. The top five most influential features were found to be the total number of medications used, the number of other medications used, the use of aspirin during pregnancy, and the use of low molecular weight heparin during pregnancy. Figure 4B represents the values of these characteristics on a spectrum, showcasing the calculated SHAP values for each characteristic in every sample. The variables are ranked in descending order by aggregating the SHAP values for each sample. For instance, a higher value for the total number of medications administered corresponds to a lower SHAP value.

### 3.7 Sample size assessment

**FIGURE 5:**
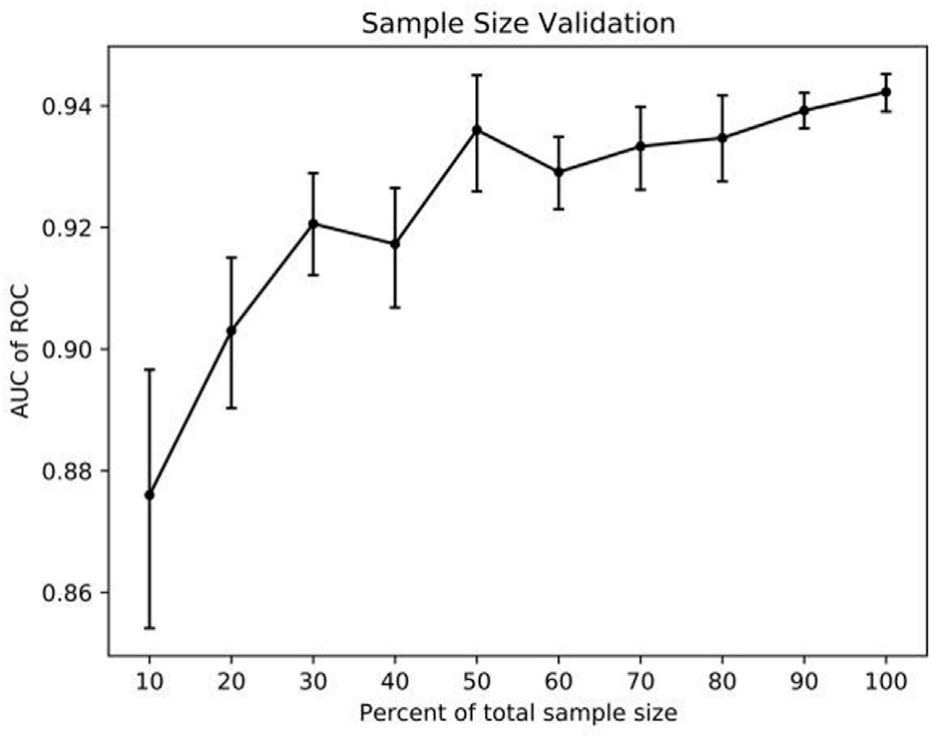
The impact of sample data size on model performances (mean ± SD).

The adequacy of the sample size was assessed using the resampling bootstrapping method, with the results displayed in Figure 5. As the size of the sample data in the model increases from small to large, a noticeable upward trend is observed in the AUC value. However, when the sample size falls within the range of 30%–60%, the curve exhibits fluctuations. Once the sample size reaches 60%, the curve tends to flatten. These findings suggest that expanding the sample size may impact the prediction model’s performance, and the model’s performance could potentially be enhanced with the addition of new samples.

## 4 Discussion

### 4.1 Principal findings

A total of 565 patients with immunologically abnormal pregnancies were included in this study. Utilizing two data sampling methods and two feature screening methods, four datasets were acquired. Subsequently, a total of 28 machine learning models were developed employing seven machine learning algorithms. The best model exhibited an AUC of 0.9209, Accuracy of 0.8469, Precision of 0.8778, Recall of 0.8061, F1 score of 0.8404, and AUPRC of 0.9395. Retrospective validation indicated the model’s overall clinical performance to be commendable.Compared to traditional maternal pregnancy risk assessment, the predictive model demonstrated enhanced performance in forecasting the likelihood of miscarriage in patients with immunologically abnormal pregnancies. Furthermore, it proved to be more user-friendly and practical for clinical implementation. An economic assessment revealed cost savings of RMB 7.48 million post-implementation, signifying the model’s economic value. The study suggested that risk prediction of miscarriage might be the most cost-effective management approach.Unlike previous studies that primarily focused on live birth or miscarriage probabilities, this research introduced a tool for predicting the likelihood of miscarriage in patients with immunologically abnormal pregnancies. This tool enables clinicians to dynamically assess a patient’s risk of miscarriage during different gestational periods, empowering them to tailor treatment based on individualized high-risk predictive factors. This personalized approach can potentially mitigate the risk of miscarriage while offering some economic relief.

In this study, we discovered that the total number of medications used during pregnancy had a positive impact on miscarriage and pregnancy complications. Conversely, the use of aspirin and low molecular heparin was associated with a reduced risk of miscarriage. Previous studies have highlighted that comorbidities during pregnancy, such as pre-eclampsia, severe vomiting, abnormal thyroid function, cholestasis, gestational diabetes mellitus, lupus nephritis, and high platelet counts, are linked to an increased risk of miscarriage (Smyth et al., 2010; Zhang et al., 2017; Turgut et al., 2022). Consequently, a higher number of comorbidities during pregnancy typically correlates with a greater risk of miscarriage. However, our study yielded a contrary conclusion, with the number of pregnancy complications showing a negative association with the risk of miscarriage. We acknowledge that a higher number of comorbidities in pregnancy is more risky in practical terms, our study came to the opposite conclusion, with the number of pregnancy complications negatively correlating with the risk of miscarriage. After discussion with clinical experts, patients with more pregnancy comorbidities received more treatment and attention during subsequent pregnancies, more frequent pregnancy follow-up, and more timely medication monitoring, which may have somewhat altered the course of pregnancy outcomes. In addition, the interactions between the diseases we analysed and the limited sample size of the study may have led to potential overfitting of the data, which could have influenced the bias given to the prediction of the outcome. Previous studies have demonstrated a connection between the breakdown of autoimmune disease tolerance and alterations in reproductive health, thereby impacting the clinical wellbeing of patients (Nielsen and Christiansen, 2005; Bowman et al., 2015). In our current study, we observed no significant variances in the indicators and outcomes between patients with autoimmune disease pregnancies and those with pregnancies characterized solely by abnormal autoantibodies through intergroup evaluation. Consequently, we inferred that the quantity of administered medications was positively associated solely with the activity of autoimmune disease in the patient. A greater number of medications signified a heightened autoimmune state during pregnancy and an increased likelihood of miscarriage.

Huang et al., 2021 utilized a machine algorithm to combine reproductive immune parameters and classify patients with recurrent pregnancy loss (RPL) into different risk categories, aiming to create a model for predicting pregnancy outcomes at various gestational periods based on genetic markers or common indicators. However, there was considerable variation in the performance of the prediction models. On the other hand, Shi et al., 2022 successfully employed adaptive simulation modelling algorithms to utilize clinical data from patients with recurrent spontaneous abortion (RSA), vitamin D levels, and thyroid function to explore optimal parameters and sub-features during support vector machine (SVM) evolution. However, the study had a relatively small sample size (*n* = 136). In comparison to our present study, Shi et al. reported superior predictive performance with an accuracy of 92.998%, Matthews correlation coefficient (MCC) of 0.92425, sensitivity of 93.286%, and specificity of 93.064%. Nevertheless, our study encompassed a larger sample size, employed a wider array of algorithms, and conducted retrospective and external validation of the model. Additionally, the integration algorithm utilized in our study aggregated the outputs of the five best models in the training model (evaluated based on the area under the curve, AUC) using the voting principle, resulting in improved predictive model performance.

In studies about the prediction of miscarriage risk in women with immunologically abnormal pregnancies, various methods have been employed to investigate high-risk factors in pregnant women. However, there are still shortcomings in clinical practice, model performance evaluation, and the practical application of prediction tools, including application complexity (Bruno et al., 2020; Benner et al., 2022; Huang et al., 2022; Macrohon et al., 2022; Hao et al., 2023; Luo and Zhou, 2023). Additionally, there is a lack of studies conducting retrospective or prospective validation of pregnancy risk prediction models and evaluating the economic aspects of these models. The findings indicate that the model holds significant clinical value. The prediction model in this study was developed based on prior research, considering the practical implementation in each healthcare institution, patient cooperation, and the scientific validity of the model’s predictive outcomes, thereby enhancing healthcare efficiency.

In conclusion, the ultimate aim of this study is to predict the risk of miscarriage in patients with immunoabnormal pregnancies and to provide assistance to clinicians in evaluating the risk of miscarriage. However, the model may not apply to the normal population because the inclusion of the population and the inclusion of the characteristics of the model mainly focus on patients with immunoabnormal pregnancies, and the parameters of the normal population in the screening of the characteristics of the normal population already did not meet the conditions of the screening were excluded, and in the subsequent steps of the data fitting and other steps, the data of the immunoabnormal population were even far from the data of the immunoabnormal pregnancy population. In addition, the features we included in the initial session and the subsequent adjustment of parameters were designed to predict risk, to prompt clinicians to judge the risk of a patient’s pregnancy and to choose whether or not to intervene in that patient, and were not designed to predict the effectiveness of the intervention. Secondly, for patients with immunoabnormal pregnancies that are assessed as high risk by this model, clinicians can intervene with aggressive pharmacological treatment and enhanced monitoring (on the one hand, monitoring for foetal developmental abnormalities for early detection and treatment, and on the other hand, monitoring for adverse drug reactions to ensure efficacy and safety of the medication), which can reduce the risk of abortion and thus save costs.

### 4.2 Limitations

This study has several limitations that need to be considered. Firstly, the entire dataset used in this study was obtained from medical centers, which may have limitations in terms of patient data. In addition, because this was a retrospective study with a relatively large patient base, dispersed residences, and most of them were not followed up in the hospital for a long period, the time of medication intervention and discontinuation were relatively difficult to count in detail and accurately, which may have biased the model performance. Therefore, further external validation using multicenter research data is necessary before clinical implementation.

### 4.3 Conclusion

This study represents a significant milestone in identifying patients at high and low risk of miscarriage during the treatment of immunoabnormal pregnancies using a model. The model is both suitable and easy to apply across various healthcare settings. We are in the process of developing a system integrated with a multidisciplinary immunopregnancy clinic, to provide clinicians with a more convenient and precise risk assessment tool. This approach aims to enhance access to accurate predictive models characterized by valuable predictors in a clinical setting. Ultimately, this will assist clinicians in diagnosing the risk of patients with immune-related pregnancies and initiating timely preventive treatment, thereby contributing to an improved prognosis for patients with immune-related pregnancies.

## Data Availability

The original contributions presented in the study are included in the article/Supplementary material, further inquiries can be directed to the corresponding authors.

## References

[B1] Bender A. R. Christiansen O. B. Elson J. Kolte A. M. Lewis S. Middeldorp S. (2018). ESHRE guideline: recurrent pregnancy loss. Hum. Reprod. Open 2018 (2), hoy4. 10.1093/hropen/hoy004 PMC6276652 31486805

[B2] Benner M. Feyaerts D. Lopez-Rincon A. van der Heijden O. W. H. van der Hoorn M. L. Joosten I. (2022). A combination of immune cell types identified through ensemble machine learning strategy detects altered profile in recurrent pregnancy loss: a pilot study. F. S Sci. 3 (2), 166–173. 10.1016/j.xfss.2022.02.002 35560014

[B3] Bowman Z. S. Wunsche V. Porter T. F. Silver R. M. Branch D. W. (2015). Prevalence of antiphospholipid antibodies and risk of subsequent adverse obstetric outcomes in women with prior pregnancy loss. J. Reprod. Immunol. 107, 59–63. 10.1016/j.jri.2014.09.052 25453202

[B4] Bratic B. Kurbalija V. Ivanovic M. Oder I. Bosnić Z. (2018). Machine learning for predicting cognitive diseases: methods, data sources and risk factors. J. Med. Syst. 42 (12), 243. 10.1007/s10916-018-1071-x 30368611

[B5] Bruno V. D'Orazio M. Ticconi C. Abundo P. Riccio S. Martinelli E. (2020). Machine Learning (ML) based-method applied in recurrent pregnancy loss (RPL) patients diagnostic work-up: a potential innovation in common clinical practice. Sci. Rep. 10 (1), 7970. 10.1038/s41598-020-64512-4 32409705 PMC7224066

[B6] Carp H. J. Selmi C. Shoenfeld Y. (2012). The autoimmune bases of infertility and pregnancy loss. J. Autoimmun. 38 (2-3), J266–J274. 10.1016/j.jaut.2011.11.016 22284905

[B7] Chengdu Daily (2022). National bureau of statistics, Chengdu survey team, Statistical bulletin of national economic and social development of Chengdu. Jinjiang, Chengdu: Chengdu Daily.

[B8] Chinese Medical Association Rheumatology Branch (2010). Diagnostic and therapeutic guidelines for systemic lupus erythematosus in the Chinese society of Rheumatology[J]. Chin. J. Rheumatology 14 (5), 342–346. 10.3760/cma.j.issn.1007-7480.2010.05.016

[B9] Chinese Medical Association Rheumatology Branch (2011). Guidelines for the diagnosis and treatment of antiphospholipid syndrome [J]. Chin. J. Rheumatology 15 (6), 407–410. 10.3760/cma.j.issn.1007-7480.2011.06.012

[B10] Chinese Society of Rheumatology of the Chinese Medical AssociationNational Clinical Research Center for Dermatologic and Immunologic DiseasesChinese Systemic Lupus Erythematosus Treatment and Research Group (2020). Guidelines for the diagnosis and treatment of systemic lupus erythematosus in China in 2020. Chin. J. Intern. Med. 59 (3), 172–185. 10.3760/cma.j.issn.0578-1426.2020.03.002

[B11] Coomarasamy A. Dhillon-Smith R. K. Papadopoulou A. Al-Memar M. Brewin J. Abrahams V. M. (2021). Recurrent miscarriage: evidence to accelerate action. Lancet 397 (10285), 1675–1682. 10.1016/S0140-6736(21)00681-4 33915096

[B12] Ford H. B. Schust D. J. (2009). Recurrent pregnancy loss: etiology, diagnosis, and therapy. Rev. Obstet. Gynecol. 2 (2), 76–83. 19609401 PMC2709325

[B13] Han S. Lanlan J. Zhou Y. Sun X. (2017). Antiphospholipid syndrome and pregnancy. Chin. J. Perinat. Med. 20 (6), 458–463. 10.3760/cma.j.issn.1007-9408.2017.06.013

[B14] Hao X. Zheng D. Khan M. Wang L. Hämäläinen T. Cong F. (2023). Machine learning models for predicting adverse pregnancy outcomes in pregnant women with systemic lupus erythematosus. Diagn. (Basel) 13 (4), 612. 10.3390/diagnostics13040612 PMC9955045 36832100

[B15] Huang C. Xiang Z. Zhang Y. Tan D. S. Yip C. K. Liu Z. (2021). Using deep learning in a monocentric study to characterize maternal immune environment for predicting pregnancy outcomes in the recurrent reproductive failure patients. Front. Immunol. 12, 642167. 10.3389/fimmu.2021.642167 33868275 PMC8047052

[B16] Huang J. Lv P. Lian Y. Zhang M. Ge X. Li S. (2022). Construction of machine learning tools to predict threatened miscarriage in the first trimester based on AEA, progesterone and β-hCG in China: a multicentre, observational, case-control study. BMC Pregnancy Childbirth 22 (1), 697. 10.1186/s12884-022-05025-y 36085038 PMC9461209

[B17] Jia E. Yaochu X. U. Shen H. Ling Z. Rongbin Y. (1999). Economic burden of disease and its evaluation method. Jiangsu Prev. Med. (03), 2–3.

[B18] Jing H. U. A. Zhu L. Li D. U. Zhuochun W. U. (2016). Effectiveness of pregnancy risk early warning assessment in improving perinatal outcomes. Chin. J. Perinat. Med. 19 (3), 200–205. 10.3760/cma.j.issn.1007-9408.2016.03.009

[B19] Khatibi T. Hanifi E. Sepehri M. M. Allahqoli L. (2021). Proposing a machine-learning based method to predict stillbirth before and during delivery and ranking the features: nationwide retrospective cross-sectional study. BMC Pregnancy Childbirth 21 (1), 202. 10.1186/s12884-021-03658-z 33706701 PMC7953639

[B20] Lakshmi B. N. I. T. S. R. (2016). A study on C.5 decision tree classification algorithm for risk predictions during pregnancy. Amsterdam, Netherlands: Elsevier Ltd, 1542–1549.

[B21] Luo Y. Zhou Y. (2023). Identification of novel biomarkers and immune infiltration features of recurrent pregnancy loss by machine learning. Sci. Rep. 13 (1), 10751. 10.1038/s41598-023-38046-4 37400532 PMC10318100

[B22] Macrohon J. Villavicencio C. N. Inbaraj X. A. Jeng J. H. (2022). A semi-supervised machine learning approach in predicting high-risk pregnancies in the Philippines. Diagn. (Basel) 12 (11), 2782. 10.3390/diagnostics12112782 PMC9689356 36428842

[B23] Malacova E. Tippaya S. Bailey H. D. Chai K. Farrant B. M. Gebremedhin A. T. (2020). Stillbirth risk prediction using machine learning for a large cohort of births from Western Australia, 1980-2015. Sci. Rep. 10 (1), 5354. 10.1038/s41598-020-62210-9 32210300 PMC7093523

[B24] National Health and Family Planning Commission of the People's Republic of China (2017). Norms for maternal pregnancy risk assessment and management. Chin. J. Pract. Rural Physicians 24 (12), 5–7. 10.3969/j.issn.1672-7185.2017.12.004

[B25] Nielsen H. S. Christiansen O. B. (2005). Prognostic impact of anticardiolipin antibodies in women with recurrent miscarriage negative for the lupus anticoagulant. Hum. Reprod. 20 (6), 1720–1728. 10.1093/humrep/deh790 15774545

[B26] Ping L. I. Yue'e Z. U. Qinzhen Y. I. Xiao Y. Liu M. Xiaolan F. A. N. (2016). Analysis of pregnancy risk early warning management in Changsha City in 2014. China Maternal Child Health 31 (06), 1136–1138. 10.7620/zgfybj.j.issn.1001-4411.2016.06.05

[B27] Practice Committee of the American Society for Reproductive Medicine (2012). Evaluation and treatment of recurrent pregnancy loss: a committee opinion. Fertil. Steril. 98 (5), 1103–1111. 10.1016/j.fertnstert.2012.06.048 22835448

[B28] Robinson G. E. (2014). Pregnancy loss. Best. Pract. Res. Clin. Obstet. Gynaecol. 28 (1), 169–178. 10.1016/j.bpobgyn.2013.08.012 24047642

[B29] Shi B. Chen J. Chen H. Lin W. Yang J. Chen Y. (2022). Prediction of recurrent spontaneous abortion using evolutionary machine learning with joint self-adaptive sime mould algorithm. Comput. Biol. Med. 148, 105885. 10.1016/j.compbiomed.2022.105885 35930957

[B30] Sichuan Daily (2022). Standing Committee of the Sichuan Provincial People’s Congress, Regulations on Population and Family Planning. Jinjiang, Chengdu: Sichuan Daily.

[B31] Singh S. McGlennan A. England A. Simons R. (2012). A validation study of the CEMACH recommended modified early obstetric warning system (MEOWS). Anaesthesia 67 (1), 12–18. 10.1111/j.1365-2044.2011.06896.x 22066604

[B32] Smyth A. Oliveira G. H. Lahr B. D. Bailey K. R. Norby S. M. Garovic V. D. (2010). A systematic review and meta-analysis of pregnancy outcomes in patients with systemic lupus erythematosus and lupus nephritis. Clin. J. Am. Soc. Nephrol. 5 (11), 2060–2068. 10.2215/CJN.00240110 20688887 PMC3001786

[B33] Subbe C. P. Kruger M. Rutherford P. Gemmel L. (2001). Validation of a modified early warning score in medical admissions. QJM 94 (10), 521–526. 10.1093/qjmed/94.10.521 11588210

[B34] Tasadduq R. Ajmal L. Batool F. Zafar T. Babar A. Riasat A. (2021). Interplay of immune components and their association with recurrent pregnancy loss. Hum. Immunol. 82 (3), 162–169. 10.1016/j.humimm.2021.01.013 33581927

[B35] Tektonidou M. G. Andreoli L. Limper M. Amoura Z. Cervera R. Costedoat-Chalumeau N. (2019). EULAR recommendations for the management of antiphospholipid syndrome in adults. Ann. Rheum. Dis. 78 (10), 1296–1304. 10.1136/annrheumdis-2019-215213 31092409 PMC11034817

[B36] Turgut E. Yildirim M. Sakcak B. Ayhan S. G. Tekin O. M. Sahin D. (2022). Predicting miscarriage using systemic immune-inflammation index. J. Obstet. Gynaecol. Res. 48 (3), 587–592. 10.1111/jog.15156 35040233

[B37] Vaulet T. Al-Memar M. Fourie H. Bobdiwala S. Saso S. Pipi M. (2022). Gradient boosted trees with individual explanations: an alternative to logistic regression for viability prediction in the first trimester of pregnancy. Comput. Methods Programs Biomed. 213, 106520. 10.1016/j.cmpb.2021.106520 34808532 PMC8674730

[B38] Writing Group Of Chinese Expert (2020). Chinese expert consensus on the diagnosis and treatment of spontaneous abortion (2020 edition). Chin. J. Pract. Gynaecol. Obstetrics 36 (11), 1082–1090. 10.19538/j.fk2020110113

[B39] Zhang H. Jiang J. X. Xia J. Yu C. Zhong M. H. Pang Q. Y. (2023). Analysis of clinical application effects and challenges of early warning system for the high-risk obstetric women of China: a scoping review. J. Clin. Nurs. 32 (9-10), 2073–2085. 10.1111/jocn.16288 35304785

[B40] Zhang Y. Wang H. Pan X. Teng W. Shan Z. (2017). Patients with subclinical hypothyroidism before 20 weeks of pregnancy have a higher risk of miscarriage: a systematic review and meta-analysis. PLoS One 12 (4), e0175708. 10.1371/journal.pone.0175708 28414788 PMC5393567

